# Engineering and Evaluation of Forcespun Gelatin Nanofibers as an Isorhamnetin Glycosides Delivery System

**DOI:** 10.3390/pharmaceutics14061116

**Published:** 2022-05-24

**Authors:** Elsy J. García-Valderrama, Narsimha Mamidi, Marilena Antunes-Ricardo, Janet A. Gutiérrez-Uribe, Karina Del Angel-Sanchez, Alex Elías-Zúñiga

**Affiliations:** 1 Tecnologico de Monterrey, Centro de Biotecnología-FEMSA, Av. Eugenio Garza Sada 2501 Sur., Monterrey C.P. 64849, Mexico; elsiiigv@gmail.com (E.J.G.-V.); marilena.antunes@tec.mx (M.A.-R.); 2 Tecnologico de Monterrey, Department of Chemistry and Nanotechnoloogy, Campus Monterrey, Escuela de Ingeniería y Ciencias, Av. Eugenio Garza Sada 2501 Sur., Monterrey C.P. 64849, Mexico; kdelangel@tec.mx (K.D.A.-S.); aelias@tec.mx (A.E.-Z.); 3 Tecnologico de Monterrey, The Institute for Obesity Research, Av. Eugenio Garza Sada 2501 Sur., Monterrey C.P. 64849, Mexico; 4 Tecnologico de Monterrey, Campus Puebla, Av. Atlixcáyotl 5718, Puebla C.P. 72453, Mexico

**Keywords:** crosslinking, isorhamnetin glycosides, forcespun nanofibers, *O. ficus-indica*, delivery system

## Abstract

*Opuntia ficus-indica* (L.) Mill (OFI) is considered a natural source of bioactive phytochemicals, mainly isorhamnetin glycosides (IRGs). These compounds have demonstrated antioxidant, anti-inflammatory, and anticancer activities, among others. The development of a suitable delivery system for these compounds is needed to improve their chemical and biological stability. This study aimed to evaluate the feasibility of fabrication and characterization of IRG-loaded gelatin (GL) forcespun fibers and crosslinking with glutaraldehyde (GTA). Two different percentages (25% and 30% *w*/*v*) of GL were evaluated with 12% (*w*/*v*) OFI flour to obtain nanofibers GL/OFI1 and GL/OFI2, respectively. The morphology and physicochemical properties of the fibers were investigated. The results indicated that the diameters of the fibers were on the nanoscale. The amount of IRGs was determined using high-performance liquid chromatography (HPLC). The IRGs release and the cytocompatibility of the nanofibers were also evaluated. GL concentration significantly affected the IRG release. Among both nanofibers, the GL/OFI2 nanofiber achieved a cumulative IRGs release of 63% after 72 h. Both fibers were shown to be biocompatible with human skin/fibroblast cells. Specifically, GL/OFI1 nanofibers exhibited favorable features for their application as an extract-coupled release system. The IRGs-embedded GL nanofiber mats may become a good alternative for the delivery of phytochemicals for the health sector and biomedical applications.

## 1. Introduction

Nowadays, nanotechnology has developed a wide range of products, such as nanoparticles [1,2], nanorods, nanosponges [3], nanogels [4,5], and nanofibers [6,7,8], as delivery systems for food, medical, and cosmetic applications. A recent application of these nanotechnology-based products is the development of composite systems in which synthetic or biodegradable polymers and plant extracts are coupled to create nontoxic, biocompatible, and functional matrixes [9,10,11,12]. Nanofibers have been a useful strategy in the development of these composite systems and scaffolds, since their similarities with skin characteristics, such as small pore size, high surface-area-to-volume ratio, high tensile strength, high permeability, low weight, and gas exchange, facilitate nutrient transport, waste excretion, and a low coefficient of thermal expansion, enhancing cell attachment, migration, and differentiation [13,14,15]. Therefore, nanofibers can act as suitable carrier platforms for synthetic or natural compounds that have poor bioavailability and are considered an effective drug release system [16,17]. Lately, natural biopolymers such as zein, silk fibroin, gelatin (GL), keratin, collagen, and elastin have attracted the interest and attention of researchers due to their biocompatibility, biodegradability, hydrophobicity, nontoxic effects on a biological system, and greater tissue regeneration rate [17,18,19]. Among them, GL is a natural biomaterial with low antigenicity compared with native collagen [20]. GL exhibits many advantages as a biopolymer for biomedical applications, including its abundancy, good oxygen barrier capacity, low gelling, and low cost [21]. However, GL nanofibers have faced the disadvantage of being dissolved in aqueous conditions, which prevents their wide range of applications in tissue scaffolds [22]. To solve this, the combination of GL with other polymer or crosslinking processes have arisen to improve the water stability and thermomechanical properties of GL-based nanofibers [23]. Crosslinking agents such as formaldehyde, glutaraldehyde, glyceraldehyde, carbodiimide, genipin, and dextran dialdehyde have been used to chemically modify the GL structure [24,25,26,27]. Glutaraldehyde (GTA) has been widely used through vapor phase crosslinking reactions [19,28]. Compared with other crosslinking agents, GTA has a proven high efficiency in collagenous-derived materials, high biodegradation, and lower cytotoxicity, creating a biocompatible material that maintains flexibility and strength [24,29]. The wide use of GTA relies on its easy availability, low cost, and effectiveness in a short period [30].

The production of nanofibers is usually primed by the electrospinning method (E-spinning) based on the electrostatic principle. E-spinning consists of driving polymer solutions through high-voltage conditions, so droplets of the solution can be ejected from a metallic needle, stretched by the influence of voltage, and then collected in a counter electrode [31,32]. However, new methods have been developed to overcome electrospinning limitations, such as low yield, the use of specialized equipment, high electrical potential, electrically conductive targets, and high costs [33,34]. Forcespinning™ (FS) or the centrifugal spinning method has emerged as a novel strategy for the fabrication of nanofibers, and this method uses centrifugal force to form ultrafine nanofibers (from 25 nm to several µm) [35]. Unlike E-spinning, the FS method consists of ejecting a polymer solution through thin needles which rotate at high-speed conditions, thus the centrifugal force produces solidification, and the stretching of the polymer and the evaporation of the solvent, allowing for the collection of the nanofibers on the walls of a cylindrical collector [36,37]. Since it uses centrifugal forces instead of electric fields, the FS method provides a higher productivity rate, over 1 mL/min per nozzle, around 200 times higher than E-spinning [35]. FS also allows the use of nonconductive and conductive polymer solutions, as well as melts, to be spun into nanofibers [38].

Herb-derived components are more effective and less toxic than conventional medicines, which makes them an excellent alternative for the production of herb-incorporated scaffolds or dressings for health purposes [31,39]. Bioactive constituents such as phenolic compounds, saponins, tannins, quinones, and flavonoids typically found in plants have been used for the fabrication of functional fibers. It is reported that different plants are rich in phenolic compounds, and they have been incorporated into fiber production [40,41,42,43]. For instance, Hani et al. [44] coupled *Moringa oleifera* leaf crude ethanolic extract rich in quercetin, kaempferol, and ascorbic acid with GL to generate nanofibers via an E-spinning method that could act as a potential oxidative stress inhibitor. Icariin (ICA), a flavonoid glycoside extracted from *Herba epidermii*, has been used to develop ICA-loaded polycaprolactone (PCL)/GL microfibers as an efficient release system to inhibit collagen and fibronectin accumulation and validate its use as a system for the prevention of scar and adhesion formation [45]. Chrysin–curcumin-loaded nanofibers have shown in vivo anti-inflammatory properties through the modulation of the inflammatory and wound-healing biomarkers, such as interleukin (IL)-6, matrix metalloproteinase-2 (MMP-2), MMP-2, metallopeptidase inhibitor (TIMP) 1 and 2, and inducible nitric oxide synthase (iNOS) gene expression [46]. Likewise, the in vivo application of astragaloside IV-loaded nanofibers has been demonstrated to promote wound healing at the early stage through a significant increase in angiogenesis, the improvement of the immune response, and the inhibition of scar formation [47]. Almasian et al. [9] evaluated the effect of polyurethane (PU)-based nanofiber wound dressings containing *Malva sylvestris* extract on the healing of diabetic wounds. Nanofibers containing 15% *w*/*w* of *M. sylvestris* extract showed antibacterial activity (>69% inhibition) against *Staphylococcus aureus* and *Escherichia coli*. In the in vivo study, the wound healing rate of the animals treated whit this PU-based nanofiber was about 95% after 14 days. It also confirmed an increased macrophage infiltration, neovascularization activity, and fibroblastic proliferation in those groups treated with this herbal wound dressing. Annatto extract-loaded cellulose nanofibers did not demonstrate skin irritability or cytotoxicity, and showed a modulatory effect in the inflammatory process, providing evidence of the potentialities of this material for wound healing [13]. Scaffolds fabricated by the combination of *Opuntia cochenillifera* mucilage extract with natural biopolymers such as chitosan and pullulan showed an important increase in proliferation of mouse embryonic fibroblast (NIH 3T3) cells after 6 days of incubation, suggesting the potential of this composite to develop novel alternatives for wound dressing products [48].

*Opuntia ficus-indica* (L.) Mill (OFI), known as nopal, has proven health benefits attributed to its dietary fiber and phytochemicals such as isorhamnetin glycosides (di and tri-glycosides) [49]. The isorhamnetin and its glycosides have shown their potential for medical applications through their involvement in different biological mechanisms such as apoptosis, adipogenesis inhibition, insulin resistance modulation, and inflammatory response [49,50,51,52,53,54]. An important challenge to incorporate these bioactive compounds into pharmaceutic or cosmetic products is their chemical stability under storage and biological conditions which directly impact their biological activity. The development of nanotechnology has allowed the development of new strategies for the stabilization of sensitive active molecules and to include them into delivery systems that can provide a controlled or sustained release of these molecules, one of these strategies is through the manufacture of nanofibers using the Forcespinning™ technology. This study aimed to evaluate isorhamnetin glycosides (IRGs)-loaded GL forcespun nanofibers crosslinked with glutaraldehyde (GL/OFI). The GL/OFI nanofibers were fabricated by using the FS technique at room temperature. The obtained GL/OFI fiber mats were characterized and cell viability and OFI release was measured under physiological conditions. The results revealed augmented cell viability and sustained as well as controlled drug release.

## 2. Methods and Methods

### 2.1. Chemicals and Reagents

Glacial acetic acid and formic acid solution (88%) were purchased from CTR scientific (Monterrey, NL, Mexico). Gelatin Type A from porcine skin in powder form, crosslinking agent of aqueous Glutaraldehyde (GTA) solution (50%), and N,N-dimethylformamide (DMF) were obtained from Sigma Aldrich (St. Louis, MO, USA). Methanol and ethanol solutions were obtained from DEQ (Monterrey, NL, Mexico). Acetonitrile (ACN) was obtained from J.T. Chemical Co. (Phillipsburg, NJ, USA). Tetrahydrofuran (THF) was obtained from Tedia Company, Inc. (Fairfield, OH, USA). Isorhamnetin standard was purchased from Indofine Chemical Co., Inc. (Hillsborough NJ, USA). HPLC-grade water and methanol were obtained from VWR International LLC (West Chester, PA, USA). Phosphate saline solution (PBS) pH 7.4 (1×) was acquired from Gibco Laboratories (Grand Island, NY, USA). Dulbecco’s Modified Medium: Nutrient Mixture F-12 (DMEM-F12) ampicillin/streptomycin and trypsin were purchased from Gibco Invitrogen (Carlsbad, CA, USA). Celltiter96^®^Aqueous One Solution Cell Proliferation Assays from Promega (Madison, WI, USA).

### 2.2. Biological Material

The *O. ficus-indica* (L.) Mill (OFI) plant was harvested in the region of Montemorelos, Nuevo León, México, and the taxonomic identification was performed at the School of Agronomy of Universidad Autónoma del Nuevo León (UANL), México. Cladodes were harvested at 7 months and processed into flour, as reported by Antunes-Ricardo et al. [55]. The flour was packaged in dark bags and stored at −20 °C until extraction. 

### 2.3. Opuntia ficus-indica Extract Preparation and Chromatographic Analysis

Before the fabrication of the nanofibers, 12% (*w*/*v*) OFI extract solution was prepared using organic solvents such as glacial acetic acid, acetic acid 80%, methanol, ethanol, ACN, THF, and DMF, separately. This is to determine the total amount of IRGs equivalents recovered per solvent. Solutions were stirred at 250 rpm for 1 h at room temperature (VWR^®^ Incubating Orbital Shaker, Model 35009I), then centrifuged at 10,000 rpm for 10 min at 4 °C (Thermo Scientific SL16R, Langenselbold, Germany). 

For chromatographic analysis, the supernatant was filtered (Whatman No. 1) using a vacuum pump and stored at room temperature. Then, 1 mL of each extract was evaporated under vacuum (Genevac™ Concentrator EZ-2 Plus HCl, Fisher Scientific, Ipswich, UK) and stored at −80 °C. The samples were resuspended using a methanol/water solution (1:5 *v*/*v*) to identify and quantify the IRGs according to the protocol by Antunes-Ricardo et al. [56]. Analysis of IRGs was performed using high-performance liquid chromatography (HPLC) (Agilent 1100 Series Santa Clara, CA, USA) with a Zorbax XDB C_18_ (4.6 mm × 150 mm, 5 µm) column at 25 °C, a flow of 0.45 mL/min, and an injection volume of 2 µL. The mobile phase consisted of (A) HPLC-grade water with 0.1% of formic acid and (B) HPLC-grade methanol. The gradient was started with 35% of B for 5 min, increasing to 60% in 15 min, and in the next 5 min, the percentage of B changed to 90%. Chromatograms were obtained at 365 nm. Standard curves for isorhamnetin were used and the IRGs were quantified as isorhamnetin equivalents (IsoEq).

### 2.4. Nanofiber Fabrication and Crosslinking Treatment

**Figure 1:**
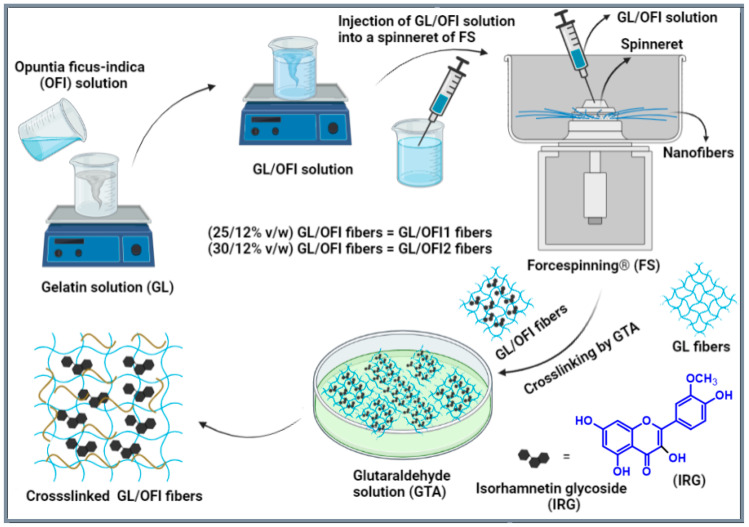
Schematic representation of the fabrication and crosslinking process of forcespun GL/OFI nanofibers.

After the exaction of IRGs from OFI, the forcespun nanofibers were fabricated by using FS followed by crosslinking with GTA at room temperature (Figure 1). Two GL solutions were prepared using different concentrations of gelatin (25% and 30% *w*/*v*), and the OFI (12% *w*/*v*) extract solution was prepared with acetic acid 80% (*v*/*v*), giving the GL/OFI1 and GL/OFI2 solutions. Each solution was mixed in the solvent and stirred at 150 rpm for 8 h at 55 °C. Then, the uniform GL/OFI solution (2 mL) was injected into a spinneret of the FS (Fiberio Fiberlab L1000). The velocity of spinneret and time for GL/OFI1 solution was 5500 rpm and 1 min, respectively. On the other hand, the velocity of the spinneret and time for GL/OFI2 solution was 9000 rpm and 1 min, respectively. Pristine GL (30% *w*/*v*) nanofibers were fabricated by the FS and used as control nanofibers. The obtained GL/OFI nanofibers were crosslinked by using GTA. Henceforth, GL/OFI1 and GL/OFI2 nanofibers nomenclature was used throughout the article. 

Nanofibers were placed in a desiccator containing 25 mL of 30% aqueous GTA solution in a Petri dish. The nanofibers were kept at room temperature for 3 days in a Nalgene desiccator. To remove excess GTA, the nanofibers were exposed to a fume hood for 1 h and then dried for 24 h in an oven at 50 °C.

### 2.5. Cell Viability Assays

Cell viability was performed using CellTiter96^®^AQueous One Solution Cell Proliferation Assay. GL/OFI nanofibers were punched to circular discs (7 mm) and, to ensure sterilization, they were exposed to UVA radiation under a laminar flow hood for 1 h and washed with PBS twice. Cells were seeded in a 96-well plate at a density of 1 × 10^4^ cells per well. After 24 h, nanofibers were immersed in each well. Then, after 24, 48, and 72 h, the viability was measured using 20 µL of CellTiter96 per well, incubating for 1 h at 37 °C, and then separating the supernatants to another 96-well plate. Absorbance was measured at 490 nm with a 96-well microplate reader (Synergy HT, BioTec, Winooski, VT, USA). Cell viability was obtained by dividing the absorbance of the cells with the OFI nanofiber by the absorbance of the control cells. 

### 2.6. Characterization of Nanofibers

The morphology of the nanofibers was observed using Scanning Electron Microscopy (SEM) (ZEISS EVO^®^ MA 25, Ostalbkreis, Baden-Württemberg, Germany) at EHT: 10.00 kV. The size distribution and the pore size of fibers were measured by the ImageJ analyzer software version 1.53 (NIH, Bethesda, MD, USA). Nanofibers were analyzed using Fourier transform infrared (FTIR) spectroscopy (Perkin Elmer Universal ATR Sampling Accessory Frontier). Thermal properties were measured by Differential Scanning Calorimetry (DSC). Infinity focus microscopy was performed using confocal microscopy Zeiss Axio CSM 700 Controller (Carl-Zeiss-Strasse 22, 73447 Oberkochen, Germany). Water contact angle measurements were performed using the Data physics (OCA, 15EC) system (Raiffeisenstraße 34, 70794 Filderstadt, Germany). The contact angle between the mesh surface and the water droplet was measured by SCA20_U Software (Version 2, DataPhysics Instruments GmbH, Filderstadt, Germany).

### 2.7. Image Analysis

Image analysis was performed by using the previous report [57].

*(a)*  *Diameter measurements*

The high-resolution SEM images (TIFF format) of the fibers were applied to ImageJ analyzer software version 1.53 (NIH, Bethesda, MD, USA) to measure the average diameter of fiber mats. Several diameter measurements were performed over 110 random locations of each micrograph and averaged together. The average diameter of fiber mats with standard deviation was assessed.

*(b)*  *Porosity measurements*

The average pore size of the fiber mates was measured by using ImageJ analyzer software version 1.53 (NIH, Bethesda, MD, USA). The high-resolution SEM micrographs in TIFF format were utilized and more than 110 different measurements were assessed for each fiber mat. Then, the recorded measurements were averaged together for each pore examination to calculate the pore diameter distribution of fiber mats.

### 2.8. Quantification of Isorhamnetin Glycosides Loaded to Nanofibers

Free and bound phenolic compounds were extracted using the method reported by Acosta-Estrada et al. [58]. The total amount of isorhamnetin glycosides (IRG) was obtained by HPLC-DAD (Agilent 1100 Series Santa Clara, CA, USA) following the Antunes-Ricardo et al. [45] protocol. Isorhamnetin glycosides were quantified as isorhamnetin equivalents (IsoEq).

### 2.9. Analysis of Isorhamnetin Glycosides Release

The released amount of IRGs was determined by suspending the nanofibers in 5 mL of PBS pH 7.4. The nanofibers were incubated at 37 °C while shaking at 100 rpm in an incubator/shaker (VWR^®^ Incubating Orbital Shaker, Model 35009I). At 0.5, 1, 2, 4, 6, 8, 12, 24, 28, and 72 h, 200 µL of the supernatant was removed from the medium and then the same volume of PBS was added. The collected samples were analyzed using the HPLC-PDA (Agilent 1100 Series, Santa Clara, CA, USA) protocol described by Antunes-Ricardo et al. [56]. Standard curves of isorhamnetin were used. The isorhamnetin glycosides release percentage was calculated by dividing the isorhamnetin glycosides amount (as IsoEq) in the supernatants at each sampling time by the total amount of isorhamnetin glycosides in the nanofibers. The drug release percentage was plotted against time.

(1) $$Release\;\left(\%\right)=\frac{Released\;IsoEq}{Total\;IsoEq}\times 100\;\;\;$$

### 2.10. Cell Culture

The human dermal fibroblast (HDFa PCS-202-012TM) cell line was obtained from American Type Culture Collection (ATCC, Manassas, VA, USA). Cells were cultured in Petri plates with Dulbecco’s Modified Eagle Medium (DMEM) supplemented with 10% fetal bovine serum (FBS) and 1% antibiotic and incubated at 37 °C and 5% of CO_2_. Cytocompatibility of *O. ficus-indica* nanofibers was evaluated to determine cell viability after 24, 48, and 72 h.

### 2.11. Statistical Analysis

Experiments were performed at least in triplicate and the results were analyzed with JMP 14.0 software (SAS Institute Inc., Cary, NC, USA) using ANOVA followed by Tukey’s HSD tests. For each data set, *p* ≤ 0.05 was considered statistically significant.

## 3. Results and Discussion

### 3.1. Analysis of Isorhamnetin Glycosides

**Figure 2:**
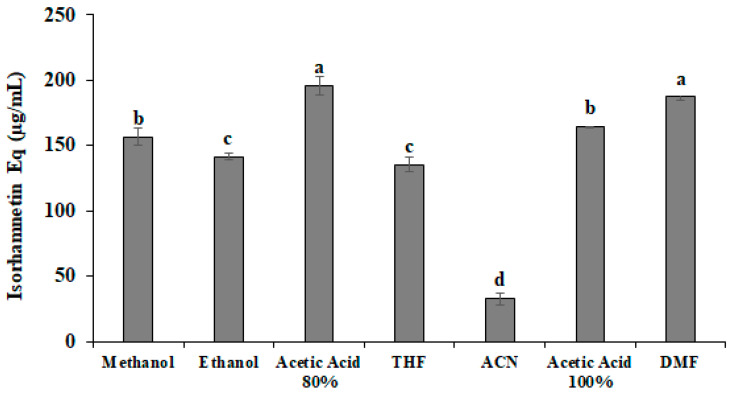
Isorhamnetin equivalents (IsoEq) in a 12% *w*/*v* of *Opuntia ficus-indica* (OFI) extract solution using different solvents. Data are shown as mean ± SD values. (a,b,c,d) Values with a different letter(s) are statistically different (HSD Tukey *p* < 0.05).

The most abundant isorhamnetin glycosides, such as isorhamnetin diglycosides: isorhamnetin-3-O-glucosyl-pentoside, isorhamnetin-3-O-glucosyl-rhamnoside, and isorhamnetin triglycosides: isorhamnetin-3-O-glucosyl-rhamnosyl-rhamnoside and isorhamnetin-3-O-glucosyl-rhamnosyl-pentoside, as well as isorhamnetin aglycone, were quantified after extract preparation and nanofiber production. Acetic acid 80% (*v*/*v*) and DMF showed the highest amount of isorhamnetin equivalents (IsoEq), with 196 µg/mL and 187 µg/mL, respectively (Figure 2). Using glacial acetic acid, 164.29 µg/mL of IsoEq was obtained. Results showed that the ethanol and THF extracts were not statistically different between them, with 142 µg/mL and 135 µg/mL of IsoEq, respectively. On the other hand, the CAN extract showed the lowest amount of IsoEq, with 33 µg/mL. Therefore, the selected OFI extract for fiber production was obtained with 80% acetic acid.

### 3.2. Characterization of GL/OFI Nanofibers

**Figure 3:**
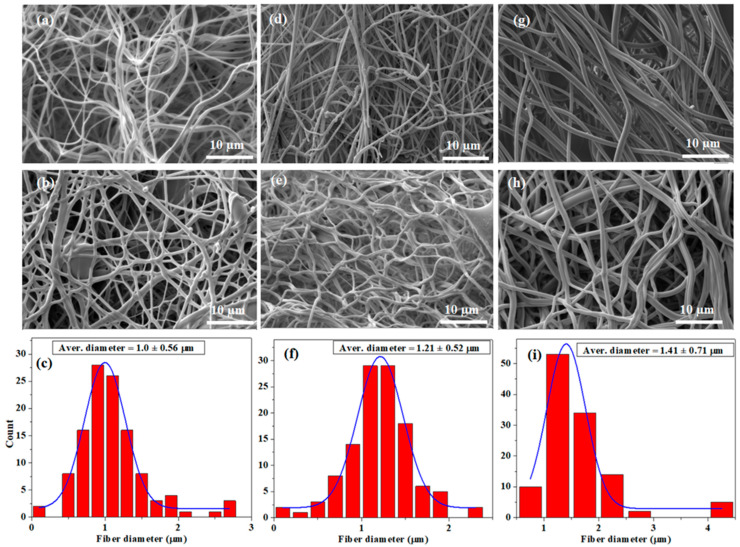
SEM images of (**a**,**d**,**g**) noncrosslinked pure GL, GL/OFI1, and GL/OGI2 fiber mates. SEM images and fiber diameter distribution of (**b**,**c**) pure GL, (**e**,**f**) GL/OFI1, and (**h**,**i**) GL/OFI2 crosslinked nanofibers, respectively.

Under the optimized conditions, pristine GL and GL/OFI fiber mats were primed by using FS. The pristine GL and GL/OFI fiber mats were crosslinked with GTA vapor. The SEM images were recorded for both noncrosslinked and crosslinked pristine GL and GL/OFI fiber mats. Noncrosslinked pristine GL, GL/OFI1, and GL/OFI2 fiber mats are presented in Figure 3a,d,g, whereas the crosslinked pristine GL, GL/OFI1, and GL/OFI2 fiber mats are shown in Figure 3b,e,h. The surface morphology of the GTA crosslinked pure GL and GL/OFI nanofibers were different from that of the noncrosslinked pure GL and GL/OFI fibers. The crosslinked fiber mats appeared to be rubberier and many fibers at the fiber hinges were fused (Figure 3b,e,h). The GTA crosslinked GL/OFI2 fiber mats better preserved their surface morphology than did GTA crosslinked pristine GL and GL/OFI fiber mates. This could be due to the slower reaction rate of GTA as compared to the pure GL and GL/OFI1 fibers, allowing the GL to dissolve before GTA could completely crosslink the fiber mats.

As illustrated in the SEM micrographs, homogeneous and beads-free interconnected forcespun nanofibers were primed, and a flat surface was observed without any drug crystals (Figure 3). The control solution prepared with GL did not produce beads and achieved an average nanofiber diameter of 1.0 µm (Figure 3b,c). The GL/OFI1 nanofibers produced homogeneous fibers with few beads and reached a nanofiber average diameter of 1.21 µm (Figure 3e,f), while the GL/OFI2 nanofibers displayed a nanofiber diameter of 1.41 µm and no visible beads within the structure (Figure 3h,i). The morphology of the GL/OFI nanofibers was significantly influenced by the GL concentration. As was expected, when the concentration of the GL increased (GL/OFI2), the elasticity of the solution was enhanced, and thus the formation of beads decreased due to the GL solution resisting the surface tension-induced bead formation. Due to the higher viscosity of the GL, the nanofiber diameter became slightly thicker.

#### 3.2.1. FTIR and DSC Measurements

**Figure 4:**
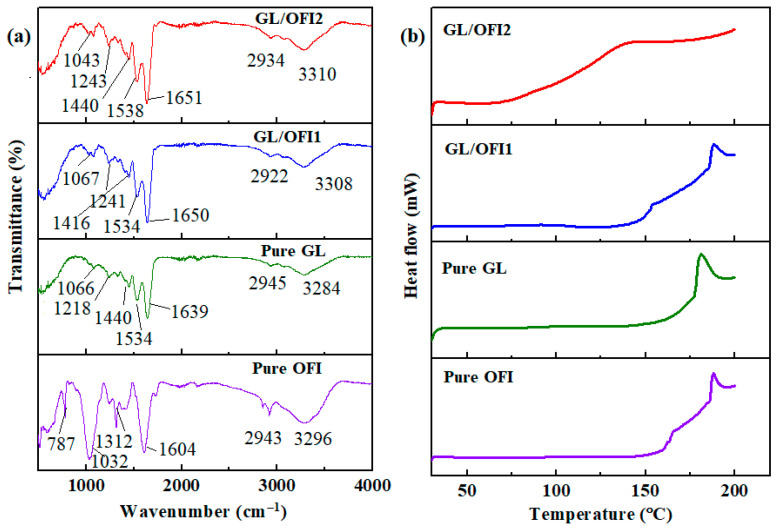
(**a**) FTIR spectra and (**b**) DSC curves of pristine GL, pure OFI, GL/OFI1 nanofiber, and GL/OFI2 nanofiber, respectively.

The GL/OFI composite fibers were characterized by using FTIR and DSC measurements, and the resulting curves are illustrated in Figure 4a. Pure GL spectra showed a characteristic band from amide A (N-H stretching vibration) and OH functional groups at 3500–3100 cm^−1^ (Figure 4a). Moreover, the absorption bands at 2945 cm^−1^ from amide B (C-N stretching vibration), at 1639 cm^−1^ from amide I (C=O stretch), and 1534 cm^−1^ from amide II were observed in the pure gelatin nanofibers [59,60,61]. The FTIR peaks of the N–H bending and C–N stretching of pure GL were observed in a range of 1440–1066 cm^−1^. The pristine OFI spectrum showed a broad absorbance band around 3296 cm^−1^, attributed to -OH stretching. An absorption band from 2960 to 2900 cm^−1^ was given by the presence of the asymmetric vibration of the -CH and -CH_2_ groups. A sharp peak at 1604 cm^−1^ is assigned to the C=C stretching vibration of a carboxylic functional group. A set of peaks in the range of 1416–1230 cm^−1^ corresponded to either H-C-H or phenolic O-H vibration. In addition, the sharp peaks at around 1312 and 1032 cm^−1^ were observed as stretching vibrations of pyranose moiety. In OFI, a peak at 787 cm^−1^ was identified as an alpha configuration of sugar moiety [62,63,64]. After incorporating the OFI solution extract into gelatin, OFI peaks at 1312–787 cm^−1^ disappeared in the FTIR spectra of the GL/OFI nanofibers, while the amide I and II peaks shifted slightly, thus confirming the electrostatic interaction or/and hydrogen bonding between the OFI and GL functional groups. Phenolic compounds, specifically flavonoids, are characterized by their noncovalent binding to proteins, since they can act as hydrogen donors to let the formation of hydrogen bonds with the functional groups of the proteins [65].

Thus, considering the chemical structure of the molecules, covalent and noncovalent interactions lead to the formation of GL-IRGs complexes. Free -OH groups present in the glycosylated flavonoids allowed the interaction with free hydroxyl groups in GL, with hydrogen bonding and π-π-interactions as the main driving forces, whereas covalent bonding by the esterification of molecules was attributed to the binding of the carboxyl groups of GL to the free -OH groups present in IRGs. Electrostatic or hydrogen bonding interactions between GL and the functional groups of IRGs were confirmed to the O-H vibration shifted to lower frequency ranges. Next, the thermal properties and decomposition stability of the pure GL, OFI flour, and the GL/OFI nanofibers were explored by DSC analysis, and the results are illustrated in Figure 4b. OFI showed a melting temperature of approximately 188 °C. Pristine GL exhibited an endothermic band at around 95 °C, which may be due to the loss of water or the denaturation of GL during the helix transition to the coil transition [60]. The second band of gelatin was observed at around 181 °C, which was the melting temperature of GL. However, the melting temperature of the GL/OFI1 and GL/OFI2 composite fibers was shifted to 189 °C. In addition, the GL/OFI2 fibers displayed a broad peak at around 150 °C, which could be due to the decomposition and oxidation of the OFI moiety. Thus, the melting temperature values of the GL/OFI composite fibers are augmented in the presence of OFI (Figure 4b).

#### 3.2.2. Infinity Focus Microscopy

**Figure 5:**
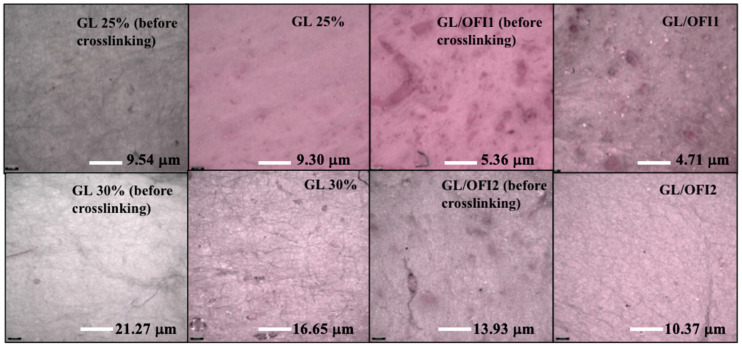
Roughness comparison of fibers with different concentrations of gelatin (GL) without or with OFI extract (GL/OFI) before and after crosslinking.

The differences in roughness between the nanofibers were observed by analyzing the values obtained for each sample, considering control composites and the GL/OFI composites with both GL concentrations before and after crosslinking. The GL/OFI2 nanofiber presented a higher roughness value (Figure 5) than the GL/OFI1 nanofibers. The GTA concentration was enough to crosslink all the GL molecules in 25% nanofibers, but it was not enough in 30% of GL. Similarly, this concept applies to the release of isorhamnetin glycosides.

#### 3.2.3. Water Contact Angle Analysis and Porosity Measurements

**Figure 6:**
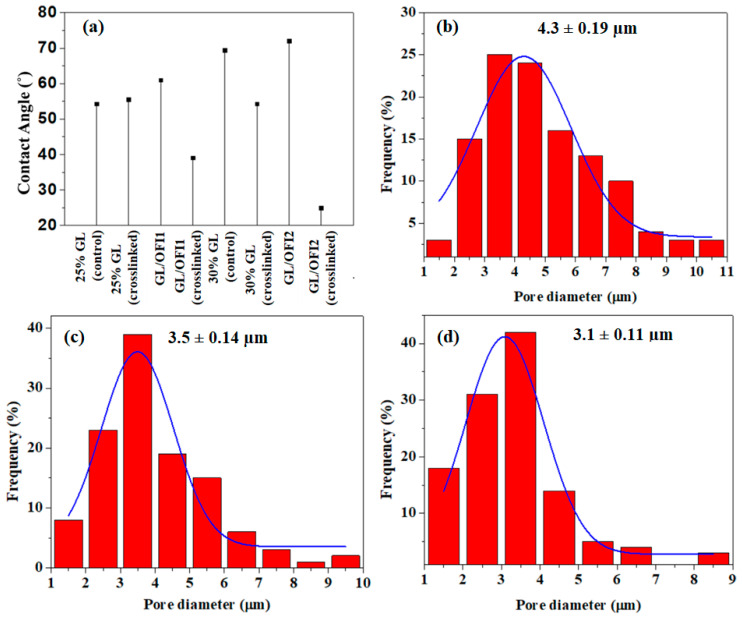
Water contact angle (CA) measurements of (**a**) forcespun pure GL, GL/OFI1, and GL/OFI2 crosslinked and noncrosslinked nanofibers. Porosity measurements of (**b**) pristine GL, (**c**) GL/OFI1, and (**d**) GL/OFI2 fiber mats.

All the fibers showed a typical GL hydrophilic nature, and it was affected by the incorporation of the OFI extract. When the 30% GL was used in combination with the OFI extract, the water contact angle was reduced in comparison to the crosslinked 30% GL, confirming that the GTA concentration used in this experiment was not enough to reduce the hydrophilic nature of the resulting fibers (Figure 6a). It is well known that the porosity of fibrous materials plays a vital role in the cellular binding study and drug release. Particularly in drug release, a large pore size would produce an initial burst drug release, whereas a small pore size would trigger a prolonged and controlled drug release phenomenon [66]. Thus, the porosity of the GL/OFI1 and GL/OFI2 forcespun fiber mats was measured and compared with the porosity of the pristine GL fiber mats (Figure 6b–d). The GL/OFI1 fiber mats exhibited 3.5 µm of average pore diameter (Figure 6c), while the GL/OFI2 fiber mats showed around 3.1 µm of average pore diameter (Figure 6d). In contrast, the pristine GL fibers showed 4.3 µm of pore diameter, which was higher than the GL/OFI fiber mats (Figure 6b). Overall, porosity measurements revealed that the porosity of the GL/OFI fiber mats was reliant on the concentration of GL. Thus, the GL/OFI2 fibers with a high concentration of GL exhibited a low porosity. The low porosity of the GL/OFI2 fiber mats may enable the prolonged and controlled OFI release from the GL/OFI fiber mats.

### 3.3. Analysis of Isorhamnetin Glycosides Release

**Figure 7:**
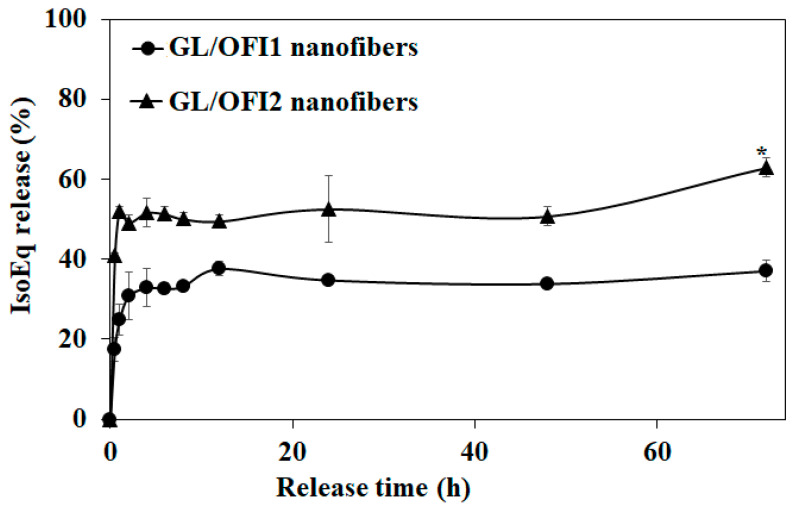
Cumulative release of isorhamnetin glycosides from FS fibers prepared with 25% or 30% gelatin and OFI extract. (*) Significant difference by Student’s *t*-test, *n* = 3 (*p* < 0.05).

A maximum cumulative isorhamnetin release of 63% and 37% was observed after 72 h from the GL/OFI2 and GL/OFI1 nanofibers, respectively (Figure 7). A low concentration of GL enhanced the interaction of carboxylic groups of GL with the free -OH groups of IRGs, thus forming nonreversible interactions such as covalent bonds. In contrast, a high gelatin concentration leads to the formation of more GTA-GL complexes, facilitating the reversible interactions between the free -OH groups of the IRGs and the hydroxyl groups of GL. After 30 min, the GL/OFI2 nanofiber released 41% of its IRG while the GL/OFI1 nanofiber had a release of only 17%. After 12 h, the release rate in both nanofibers did not change significantly, thus assuming stability. The low release of the GL/OFI1 nanofiber was related to the glutaraldehyde crosslinking treatment, which provided stability to the nanofiber structure by the glutaraldehyde carbonyl group (C=O) bonding with the free amino groups of lysine or the hydroxylysine amino acid residues of gelatin and the free OH groups from IRGs. In this sense, Sedghi et al. [67] reported a release of 89% and 67% after 10 days in chitosan (CS)/PVA nanofiber loaded with curcumin (CUR) and crosslinked using (3-aminopropyl)triethoxysilane (APTES)-modified graphene oxide. In contrast, Motealleh et al. [68] demonstrated a cumulative release rate of ~70% after 10 h from the noncrosslinked chamomile extract-loaded PCL nanofibers. 

**Figure 8:**
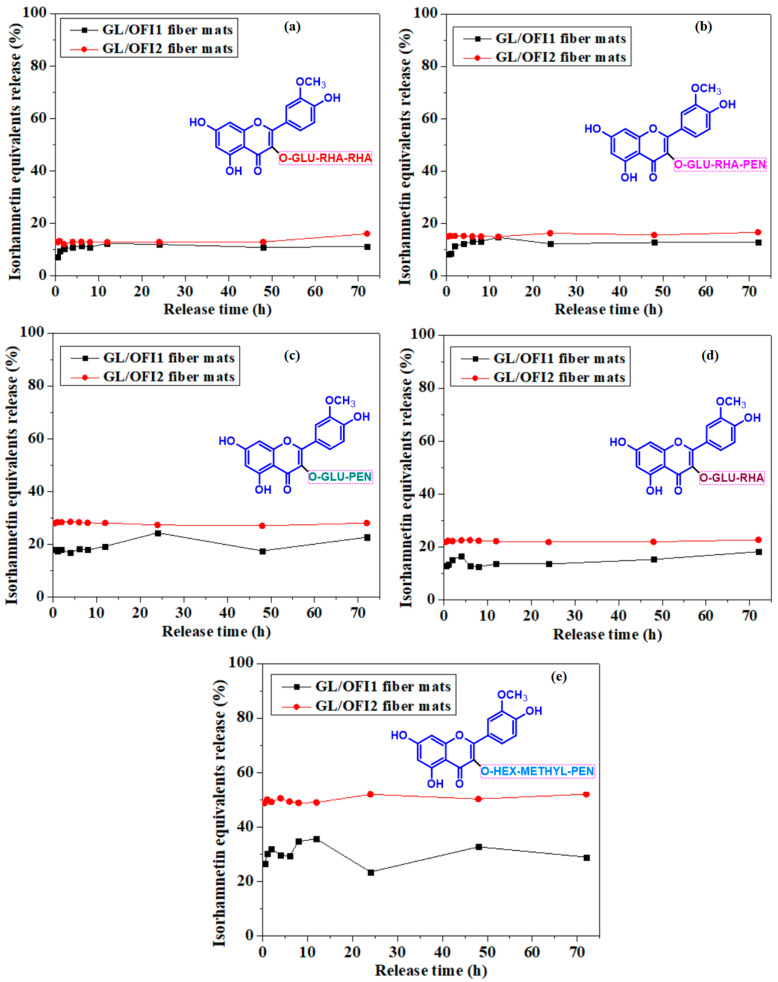
Release kinetics of different isorhamnetin glycosides: (**a**) IGRR, (**b**) IGRP, (**c**) IGP, (**d**) IGR, (**e**) IHMP] from GL/OFI1 or GL/OFI2 nanofibers. **GLU**: Glucose; **RHA**: Rhamnose; **PEN**: Pentose; **HEX**: Hexose; **MP**: Methyl pentose.

The glycosylation pattern of the IRGs also affected the established IRGs–GL chemical interactions and the release rate of these compounds from the nanofibers (Figure 8). Xiao et al. [69] and Buitimea-Cantúa et al. [70] reported the effect of the glycosylation pattern on the affinity to proteins. They established that monoglycosides enable a stronger affinity with milk proteins than complex glycosides moieties, attributing this affinity weakness to the nonplanar structure and steric hindrance of the glycoside. Alike, Martini et al. [71] reported a higher affinity to Bovine Serum Albumin (BSA) by quercetin than by quercetin 3-O-β-D-glucopyranoside. However, quercetin affinity to milk protein has been reported as 1.02 times higher than quercitrin and 54.95 times higher than rutin [69]. 

IHMP presented the highest release percentage among the other diglycosides (IGP and IGR) in GL/OFI2. In both OFI/GL fibers, diglycosides were more easily released from the matrix than IGRR and IGRP. It is suggested that IRGs–GL interactions were mainly through noncovalent bonding between the free hydroxyl groups of the GL and those free -OH groups present in the glycosidic chains of isorhamnetin.

### 3.4. Biocompatibility Assessments of GL/OFI Nanofibers

**Figure 9:**
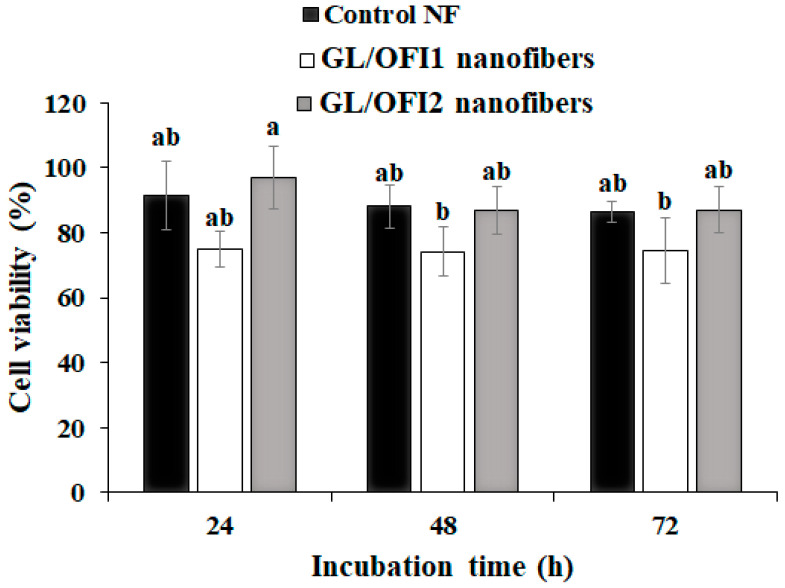
Viability of HDFa PCS-202-012TM cells in presence of GL/OFI1 and GL/OFI2 nanofibers. Data are shown as mean ± SD values. (a,b) Different letters indicate statistical differences between incubation times by each nanofiber using the Tukey test (*p* < 0.05).

No significant effect on cell viability was observed after 24 h of incubation with the GL/OFI nanofibers (Figure 9). After 48 h, the GL/OFI1 nanofibers exhibited a cell viability rate of 75% or lower, even if the amount of IRG released was significantly lower than in GL/OFI2. 

## 4. Conclusions

Fabrication of OFI-embedded GL nanofibers by the Forcespinning^®^ technique displayed an effective method to ensure the incorporation of the isorhamnetin glycosides. The manufactured nanofibers presented different average diameters and bead formation depending on the GL concentration of the solution. In contrast to GL/OFI1, the GL/OFI2 nanofiber demonstrated a thicker diameter, high elasticity, and almost null bead formation. Triglycosides were less easily released from the matrix, especially at a low GL dose, due to the interaction between IRG and the protein. GL/OFI2 presented a cumulative isorhamnetin glycosides release of 63% after 72 h. In addition, GL/OFI fibers exhibited improved cell viability against the human fibroblast cell lines. Overall, a novel drug delivery system was demonstrated in the bioinspired GL-based controlled drug delivery platform, which may be suitably useful in the future to deliver phytochemical drugs in a controlled fashion with an emphasis on tissue regenerative medicine or wound healing.
